# Hypovitaminosis and its association with recurrent aphthous stomatitis: a comprehensive review of clinical correlations and diagnostic considerations

**DOI:** 10.3389/froh.2025.1520067

**Published:** 2025-01-28

**Authors:** Alessio Rosa, Giovanni Cianconi, Riccardo De Angelis, Alberto Maria Pujia, Claudio Arcuri

**Affiliations:** ^1^ Department of Chemical Science and Technologies, Dentistry, University of Rome Tor Vergata, Rome, Italy; ^2^ Department of Biomedicine and Prevention, University of Rome Tor Vergata, Rome, Italy; ^3^ Department of Clinical Sciences and Translational Medicine, University of Rome Tor Vergata, Rome, Italy

**Keywords:** hypovitaminosis, aphthous ulcers, vitamin deficiency, recurrent aphthous stomatitis, oral health

## Abstract

**Background:**

Hypovitaminosis, or vitamin deficiency, has been increasingly recognized as a potential contributing factor in the development of recurrent aphthous stomatitis (RAS), a condition characterized by the periodic formation of painful ulcers in the oral mucosa.

**Materials and methods:**

This mini review includes a literature search on PubMed, Web of Science, and Scopus databases using keywords “hypovitaminosis AND aphthous ulcers.”

**Results:**

There is a growing body of evidence supporting the link between various vitamin deficiencies—particularly vitamins B12, C, and folate—and the prevalence of RAS, with implications for both diagnosis and management.

**Conclusion:**

This review aims to outline the clinical and biochemical findings associated with hypovitaminosis in individuals presenting with RAS, emphasizing the diagnostic importance of recognizing vitamin deficiencies in these patients and exploring possible therapeutic approaches.

## 1 Introduction

Recurrent aphthous stomatitis (RAS) is a common oral mucosal disorder presenting as painful, recurrent ulcerations of the oral cavity. RAS lesions typically appear as multiple, small, shallow, irregular ulcers with well-defined borders surrounded by erythematous haloes. While the etiology remains multifactorial, an emerging area of study highlights the role of hypovitaminosis in RAS, particularly deficiencies in vitamins B12, C, and folate. Etiological factors such as gastrointestinal malabsorption, systemic diseases, and dietary insufficiencies can impair vitamin absorption, contributing to hypovitaminosis. Vitamin deficiencies can compromise mucosal integrity, impacting cellular repair and immune response (1–3). Recognition of hypovitaminosis as a possible underlying factor in RAS offers a valuable diagnostic and therapeutic opportunity for clinicians involved in patient care.

To definitively diagnose hypovitaminosis and differentiate overlapping clinical features of vitamin deficiencies, laboratory investigations and biochemical analyses are essential. For vitamin B12 deficiency, serum vitamin B12 levels (<200 pg/ml diagnostic, 200–300 pg/ml requiring further testing), elevated methylmalonic acid (MMA), homocysteine levels, and complete blood count (CBC) findings such as macrocytosis and hypersegmented neutrophils are critical (4). Testing for intrinsic factor and parietal cell antibodies aids in identifying pernicious anemia as a cause. For vitamin C deficiency, plasma ascorbic acid levels (<0.2 mg/dl diagnostic) and erythrocyte antioxidant capacity provide reliable markers. Folate deficiency diagnosis involves serum folate (<3 ng/ml), red blood cell (RBC) folate levels (<140 ng/ml), and elevated homocysteine levels, with normal MMA helping differentiate from B12 deficiency (5–9). A comprehensive approach, including simultaneous measurement of B12, folate, and vitamin C, along with advanced tools like genetic testing for metabolic polymorphisms (e.g., MTHFR for folate metabolism), ensures accurate diagnosis and effective management. This multifaceted strategy prevents reliance solely on clinical findings and supports tailored treatment plans for conditions like recurrent aphthous stomatitis (RAS) (10–12).

The role of vitamin B12, vitamin C, and folate in the etiopathogenesis of recurrent aphthous stomatitis (RAS) can be detailed by exploring their biological functions, contributions to oral mucosal health, and involvement in metabolic pathways. Vitamin B12 is crucial for DNA synthesis, red blood cell formation, and maintaining neuronal health. Its deficiency impairs cellular repair and mucosal integrity, leading to increased susceptibility to RAS. B12 is metabolized through the cobalamin pathway, requiring gastric intrinsic factor for absorption, and is primarily excreted through bile (13–15). Disruptions in these processes, such as in malabsorption syndromes or pernicious anemia, can exacerbate deficiencies. Vitamin C plays a vital role in collagen synthesis and tissue repair by acting as a cofactor for prolyl and lysyl hydroxylase enzymes, which stabilize collagen structure. Deficiency impairs wound healing, increases mucosal fragility, and contributes to the development of painful ulcers associated with RAS (16, 17). Vitamin C is absorbed in the small intestine via active transport and is excreted through urine, with renal reabsorption maintaining physiological levels. Folate (Vitamin B9) is essential for nucleotide synthesis, DNA repair, and methylation processes, supporting rapid cell turnover in the oral mucosa. Folate deficiency disrupts epithelial cell renewal and compromises mucosal integrity, predisposing individuals to RAS (18). Folate undergoes hepatic metabolism into its active form, tetrahydrofolate, and is primarily excreted through urine. Understanding these vitamins' metabolic pathways and their excretion dynamics helps clarify their roles in RAS pathogenesis and guides targeted therapeutic interventions to restore mucosal health and prevent recurrence (19).

## 2 Clinical signs and symptoms

1. Vitamin B12 Deficiency:
  ○ Glossitis with mucosal atrophy
  ○ Increased mucosal sensitivity and erythema
  ○ Painful ulcerative lesions of the oral mucosa
2. Vitamin C Deficiency:
  ○ Delayed wound healing
  ○ Gingival bleeding and friability
  ○ Higher prevalence of small, painful ulcers
3. Folate Deficiency:
  ○ Mucosal pallor
  ○ Erosions and ulcerations along with a burning sensation
  ○ Increased risk of developing RAS in immunocompromised individuals

Therapeutic approaches for addressing hypovitaminosis should be discussed in detail, emphasizing adequate dosage, frequency, duration, and alternative delivery methods, tailored to the specific vitamin deficiency and the patient's stage of life. For vitamin B12 deficiency, oral supplementation (500–1,000 mcg daily) or intramuscular injections (1,000 mcg weekly for 4–6 weeks, followed by monthly doses) are recommended, with higher doses for severe deficiencies or absorption disorders such as pernicious anemia. Maintenance dosing typically includes 1,000 mcg monthly via injection or 1,000 mcg orally daily. For vitamin C deficiency, 100–500 mg daily is effective for mild cases, increasing to 1,000–2,000 mg daily for severe deficiencies, with gradual tapering to 100–200 mg daily for maintenance. For folate deficiency, 400–800 mcg daily is recommended, increasing to 1–5 mg daily for severe deficiencies or during pregnancy, with maintenance doses adjusted to 400 mcg daily. Adjustments based on Recommended Dietary Allowances (RDAs) should be provided for specific life stages: infancy, childhood, adulthood, pregnancy, and old age. For example, pregnant women require higher folate doses (600–800 mcg daily), while older adults may need increased B12 due to reduced absorption. Alternative delivery options, such as fortified foods or intramuscular/intravenous administration for severe cases, should be explored. This comprehensive therapeutic strategy ensures effective treatment and maintenance of adequate vitamin levels across varying needs and life stages (20).

Biochemical analysis findings, such as serum vitamin concentrations, provide valuable insights into the severity of hypovitaminosis and its association with RAS. Diagnostic workups should incorporate these findings to ensure accurate assessments, particularly in patients unresponsive to standard therapies (21–25).

## 3 Materials and methods

The PICO question was defined as follows:

• Population (P): Patients with hypovitaminosis
• Intervention (I): Measurement of vitamin levels and RAS prevalence
• Comparison (C): Patients without vitamin deficiencies
• Outcomes (O): Incidence and severity of RAS

A review of literature search was conducted on PubMed, Web of Science, and Scopus using the Boolean AND connector: “hypovitaminosis AND aphthous ulcers” (“hypovitaminosis”[MeSH Terms] OR [“hypovitaminosis”(All Fields)] OR “vitamin deficiency”[All Fields] AND [“aphthous”[All Fields] AND “ulcers”[All Fields]]. Abstracts were screened for relevance by three independent reviewers, with relevant articles selected for data extraction. Results from each database were presented separately in a tabulated format for clarity.

## 4 Results

The literature search yielded four studies meeting the inclusion criteria Table 1:

1. Tabel et al.: Case-control study on 40 individuals with documented RAS revealed 75% of participants exhibited deficiencies in vitamin B12 and folate levels. Clinical findings included glossitis, pale mucosa, and recurrent ulcerative lesions, significantly more frequent among patients with lower serum vitamin levels (26).
2. Padayatty et al.: Cohort of 25 patients with RAS reported vitamin C deficiency significantly associated with delayed ulcer healing and increased gingival bleeding. This study supports the role of vitamin C in mucosal repair and integrity, suggesting supplementation may aid ulcer resolution (26).
3. Hugar et al.: Observational study highlighted the association between folate deficiency and RAS severity. Patients with low serum folate levels showed more frequent and severe RAS lesions compared to those with normal folate levels, underscoring the importance of folate in cellular repair processes (27).
4. Freitas et al.: Evaluated the effects of vitamin supplementation on RAS resolution and observed a marked reduction in ulcer frequency among patients receiving vitamins B12, C, and folate. This finding suggests that vitamin supplementation may play a therapeutic role for RAS patients (24).

**Table 1:** Table of included studies.

Study	Year	Vitamin deficiency	Key findings	Therapeutic implications
Tabel et al.	2020	B12, Folate	Increased ulcer frequency; mucosal sensitivity	Importance of vitamin testing
Padayatty et al.	2021	C	Delayed healing; gingival bleeding	Supplementation recommended
Hugar et al.	2017	Folate	Severe RAS linked to folate deficiency	Folate therapy for severe cases
Freitas et al.	2019	B12, C, Folate	Reduction in ulcer frequency with supplementation	Multivitamin approach

## 5 Discussion

Evidence increasingly supports hypovitaminosis as a critical factor in RAS pathogenesis. Key points for consideration include:

1. Vitamin Pathways and Genetic Influences:
  ○ Research into vitamin metabolic pathways and genetic predispositions may clarify the mechanisms predisposing individuals to mucosal breakdown.
2. Innovative Diagnostic Tools:
  ○ Non-invasive serum vitamin assessments and genetic screenings could aid early detection and improve treatment outcomes.
3. Longitudinal Data:
  ○ Long-term studies assessing vitamin supplementation's impact on RAS recurrence are needed. These studies should explore optimal dosages, duration, and frequency.
4. Therapeutic Approaches:
  ○ Supplementation strategies must specify adequate dosage, frequency, and duration. Alternatives such as fortified foods or intramuscular injections for severe deficiencies warrant discussion.
5. Multidisciplinary Collaboration:
  ○ Collaboration among nutritionists, dentists, and oral medicine specialists ensures comprehensive care, from diagnosis to management (28).

## 6 Stanley's classification of RAS

**Figure 1:**
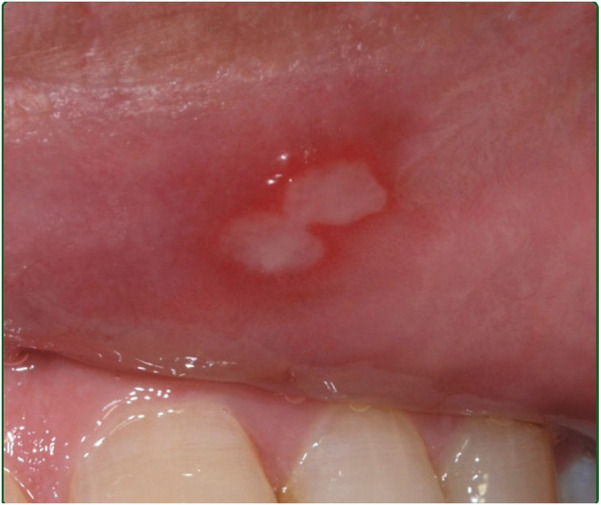
Minor RAS.

**Figure 2:**
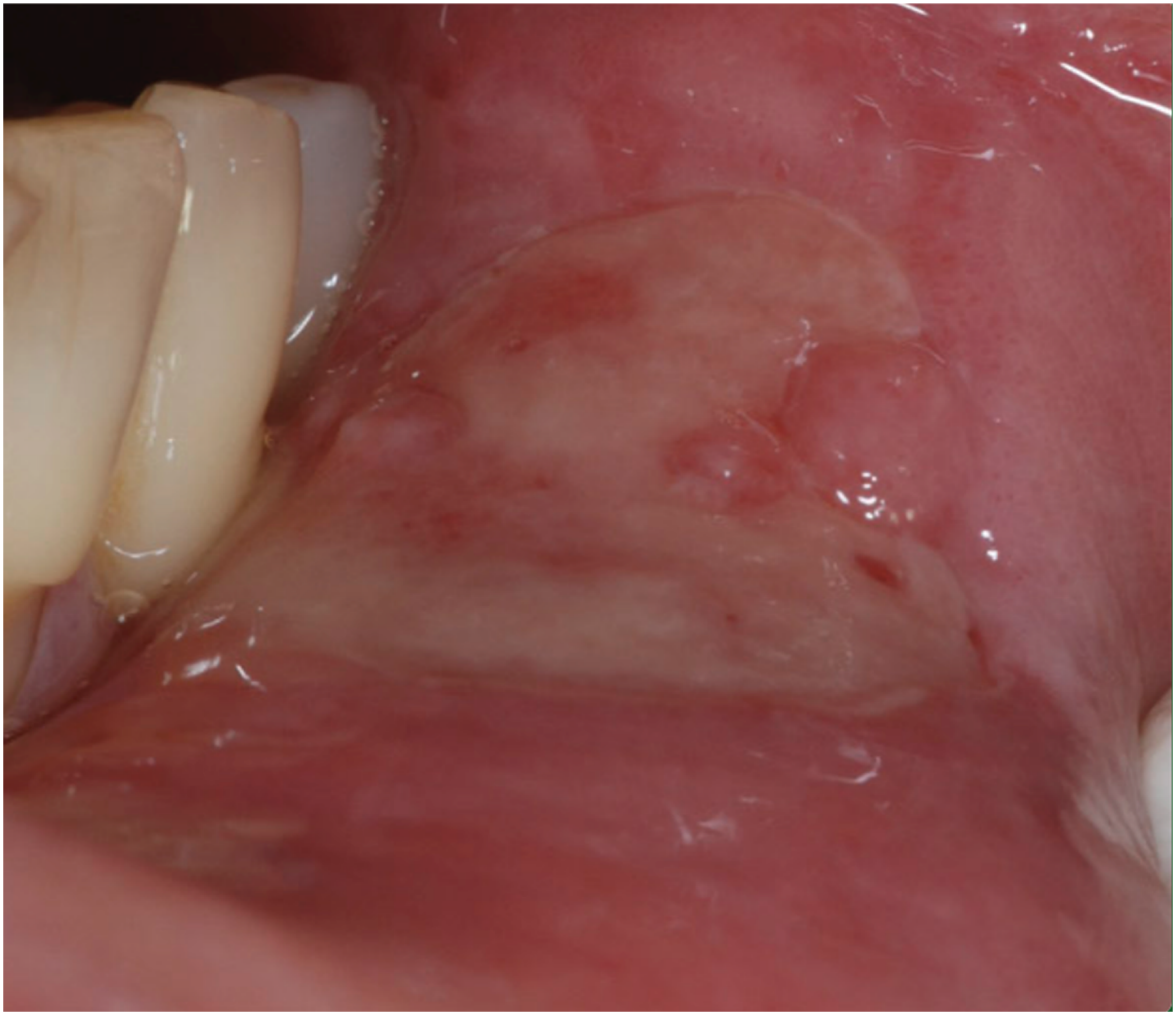
Major RAS.

**Figure 3:**
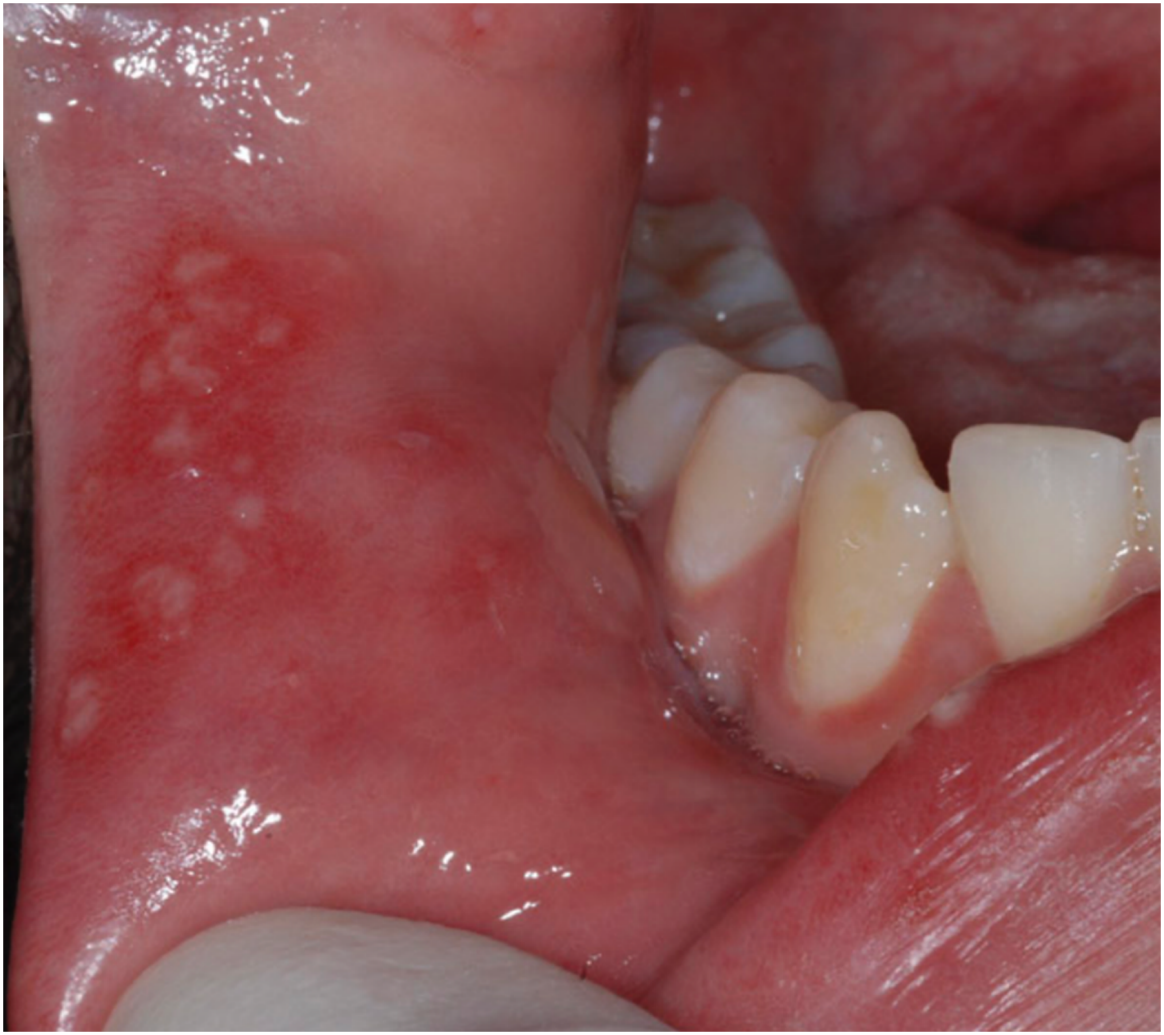
Herpetiform RAS.

RAS is classified into three main types according to Stanley's classification:

1. Minor RAS Figure 1:
  ○ The most common type, accounting for approximately 80% of cases.
  ○ Lesions are small (<10 mm), round or oval, and shallow with erythematous borders.
  ○ Typically heal within 7–14 days without scarring.
  ○ Common sites include the non-keratinized mucosa such as the inner cheeks and lips (29).
2. Major RAS Figure 2:
  ○ Less frequent, accounting for around 10%–15% of cases.
  ○ Lesions are larger (>10 mm), deeper, and more painful.
  ○ Healing may take weeks to months and often results in scarring.
  ○ Frequently observed on the soft palate, tonsillar fauces, and lips (30, 31).
3. Herpetiform RAS Figure 3:
  ○ The least common type, comprising 5%–10% of cases (5, 20).
  ○ Characterized by numerous small (1–3 mm) ulcers that can coalesce to form larger irregular lesions.
  ○ Lesions may occur on both keratinized and non-keratinized mucosa.
  ○ Heals within 7–10 days without scarring.

## 7 Conclusion

Hypovitaminosis represents a critical factor in the etiology and management of RAS. Understanding the multifactorial etiology of RAS, including gastrointestinal and systemic conditions, can guide diagnostic efforts. Identification of vitamin deficiencies, particularly involving vitamins B12, C, and folate, is essential for formulating effective diagnostic and therapeutic strategies. Further research into advanced diagnostic tools, genetic influences, and long-term supplementation protocols will enhance clinical approaches and outcomes. Multidisciplinary management remains key to optimizing care for individuals affected by this challenging oral condition.
